# Supplementary material for: Methylation of Salmonella Typhimurium flagella promotes bacterial adhesion and host cell invasion
Source: Nat Commun. 2020 Apr 24;11:2013. doi: 10.1038/s41467-020-15738-3 (PMC7181671; doi:10.1038/s41467-020-15738-3)
Supplement: Supplementary file 1 — Supplementary Information [file 41467_2020_15738_MOESM1_ESM.pdf]

Supplementary Information:

Methylation of *Salmonella* Typhimurium flagella promotes bacterial adhesion and host cell invasion

Horstmann et al.

Supplementary Notes 1–2
Supplementary Figures 1–10
Supplementary Tables 1–3
Supplementary References

Supplementary Notes:

Supplementary Note 1: Determining the structure of the flagellin FljB

Large parts of the different flagellin variants are conserved. In particular, the N- and C-termini of flagellin that form the D0 and D1 domains are highly conserved and necessary for formation of a coiled-coil structure and flagellin polymerization[1–3]. The D2 and D3 domains that form the horizontal arm of the molecule are surface-exposed in the assembled filament structure. These domains are highly variable and immunogenic, leading to recognition by host receptors and triggering of innate and adaptive immune responses, and the structural differences may also contribute to the distinct motility behavior[4–6]. However, while the structure of the F41 fragment of FliC, missing the D0 domain, has been determined previously[7], the structure of FljB remained unknown.

Accordingly, we determined the crystal structure of FljB from *S. enterica* (Supplementary Table 1). Despite several attempts, we were not able to crystallize full-length $FljB_{1-506}$ most likely due to the spontaneous polymerization caused by the D0 domain. We therefore crystallized a FljB fragment lacking the D0 domain based on the crystal structure of the F41 fragment of *Salmonella* FliC[7]. Deletion of the N- and C-terminal regions (comprising residues 1-54 and 463-506, respectively) of the D0 domain yielded crystals diffracting X-rays to 2.0 Å resolution. The crystallographic asymmetric unit contains a single copy of FljB comprising wildtype residues 55-459 in a continuous polypeptide chain and an additional serine residue in position 54 belonging to the tag inserted for affinity purification.

FljB can be divided into three domains named D1, D2 and D3 according to the structure (Fig. 1a). The topology of the FljB domains is conserved compared with FliC (Fig. 1b)[7]. Both the N- and C-termini contribute to the domain D1 with the polypeptide chain segments 55-177 and 414-459. D2 is also formed by two discontinuous segments of the polypeptide chain (residues 178-190 and 288-413) followed by domain D3 that is most distant from the FljB termini (residues 191-287) (Fig. 1b+d).

The tertiary structure of FljB resembles, similar to FliC, a boomerang with one arm formed by the D1 domain and the other formed by D2 and D3 (Fig. S2a). The major difference between the structures of the two flagellins is the rotation of the D3 domain of about 90° around the axis defined by the D2-D3 arm (Fig. S2c), resulting in the widening angle of about 20° between the two boomerang's arms. The secondary structure of D1 is however, conserved between FljB and FliC (Fig. S2b). Similarly to FliC, it is composed by a long coiled coil flanked by another helix and a β-hairpin which, together with the D0 domain, form the backbone of the flagellar filament. The sequence identity for this domain is 97 % and the Cα traces superpose well with a low r.m.s.d of 0.63 Å for residues 58-177 and 414-458. In domain D2, a short α-helix is formed by FljB residues 309-314, which is not present in FliC. The lower sequence identity for the D2 domain (64%) is reflected by the higher r.m.s.d. of 1.49 Å for the superposition of 123 matching residues. The domain D3 has the lowest sequence identity (30%) and also the greatest r.m.s.d of 1.93 Å, calculated on 87 matching residues, although its topology is well conserved between FljB and FliC (Fig. S2b). Residues 244-249 at one end of the FljB molecule form a short 3_{10} helix not present in FliC.

Supplementary Note 2: Potential role of different methylation patterns found in FliC and FljB

In FliC, 16 out of 28 lysine residues and in FljB, 18 out of 30 lysine residues are methylated. All methylation sites were found monomethylated, with the exception of FljB-K396 that was detected dimethylated only. FliC-K216, K349, K358 and FljB-K299, K341, K357 and K369 were also detected dimethylated. FliC-K293, K309 and FljB-K299, K341 and K414 are methylated to a lesser extent also in the absence of FliB (*ΔfliB*). Among the 18 lysine residues conserved between the two flagellins, we found that 10 are methylated in both flagellins, six are not methylated in either flagellin, and two are methylated in one flagellin but not in the other. The different methylation patterns found in FljB compared to FliC appear not to play an important role in the ability of the bacteria to adhere to MODE-K cells. Further, the surface hydrophobicity of the respective methylated flagellar filaments appears similar. However, FljB-expressing bacteria are less effective in invading epithelial cells, which has been attributed before to a distinct near-surface swimming behavior of FljB-expressing bacteria[8]. The different orientation of the D3 domain in the two flagellin molecules might in turn modify the shape and the physical-chemical and hydrodynamic properties of the flagellar filament bundle and thus explain the observed distinct motility behavior. As mentioned above, FliC and FljB displayed different methylation patterns and no clear consensus sequence was found for the methylation sites. The observed preferences for small amino acids in the vicinity of the methylation site might favor exposure of the methylated lysines to the epithelial cell surface by avoiding potential sterical interference of bulky side chains. The local amino acid sequence alone, however, does not seem to play a crucial role in this process since most of the methylated, conserved lysines were modified in both fully conserved domains (D0 and D1) and in domains (D2 and D3) with low sequence conservation (Fig. 1d). Therefore, the methylation seems not to be influenced significantly by changes in the flagellin local structure. This observation suggests that the flagellin methylation happens after at least partial folding of FliC or FljB, since the methylation pattern are structure related and mostly found on surface-exposed lysines (Fig. 2a).

Supplementary Figures:

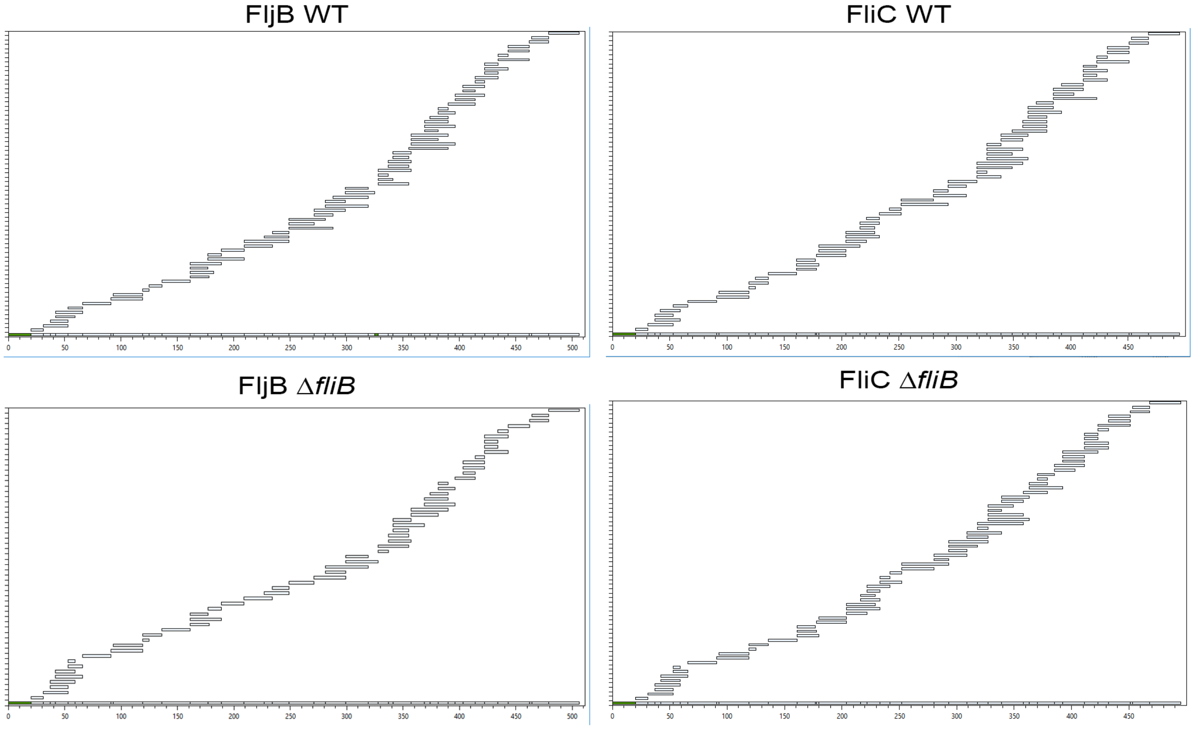

Supplementary Figure 1: Sequence coverage maps of mass spectrometry protein methylation studies. Tryptic peptides that were identified in two of three runs were plotted against the sequence of FljB (left) and FliC (right) for WT (top) and Δ*fliB* samples (bottom). For all proteins the N-terminal sequence (amino acids 1-20) was not covered. Also, amino acids 326-328 in FljB WT are missing. All the other sequence areas are identified, often in multiple peptides, resulting in coverages above 95%. Coverage maps were created using PatternLab software[9].

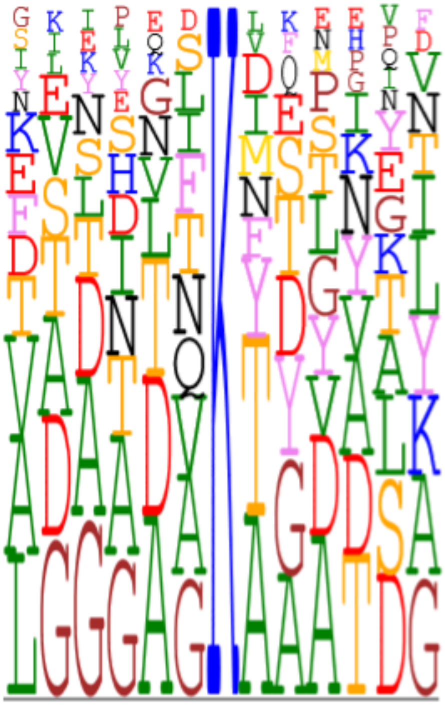

Supplementary Figure 2: **MultiDisp representation of the alignment of methylation sites found in FljB and FliC.** MultiDisp (http://structure.bmc.lu.se/MultiDisp) was used to analyze the conservation of methylation sites (methylated lysine ± 6 residues) in FljB and FliC. The occurrence of the amino acids is proportional to the dimension of the letters.

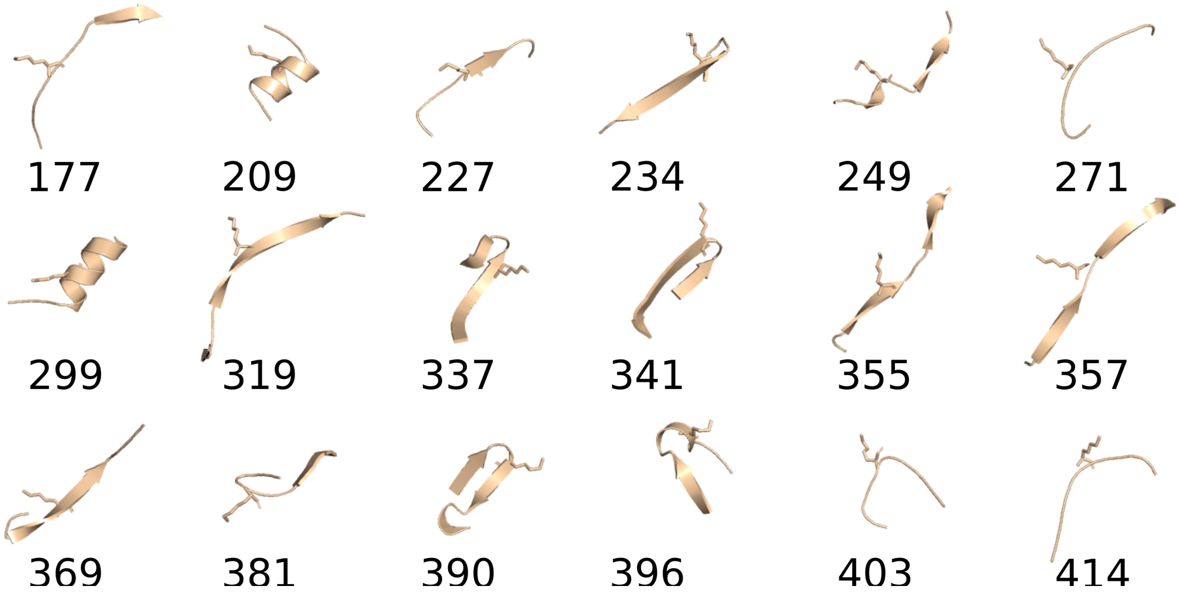

Supplementary Figure 3: Local structure of the FliB-dependent methylation sites of FljB. Secondary structure representation of the 18 identified methylation sites in FljB showing each 13 residues with the methylated lysine as stick in the center. The sequence number of the lysine is indicated below. Three methylation sites are located in a α-helix, four in a β-strand, four in a β-hairpin and seven in a loop.

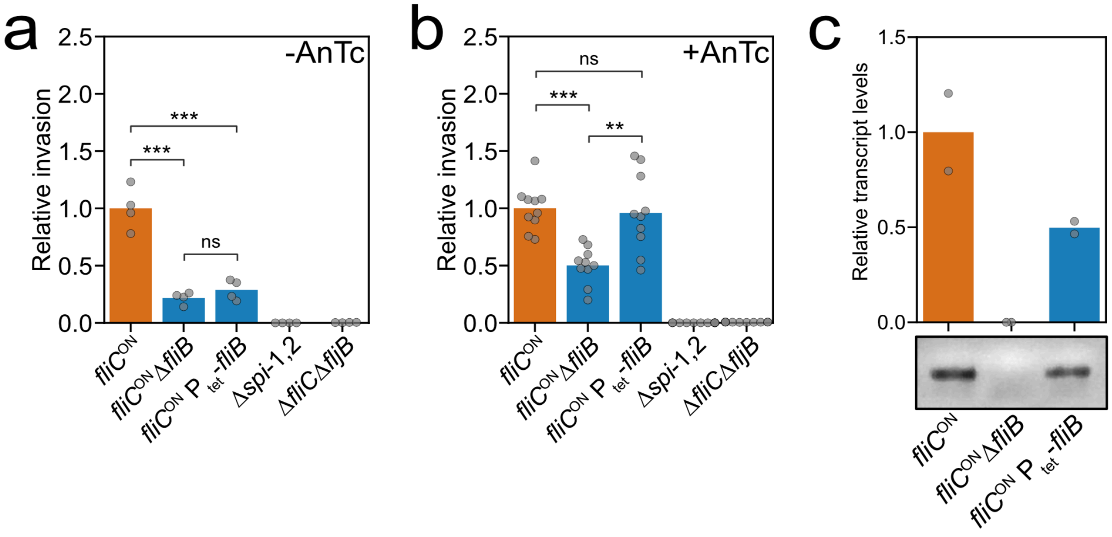

Supplementary Figure 4: Complementing the flagella methylation-dependent invasion phenotype by inducible expression of *fliB*. (a-b) Cell invasion was complemented using an inducible P_{tet} promoter chromosomally fused to *fliB* (EM5080). FliB expression was induced by addition of 100 $ng{\cdot}mL^{-1}$ anhydrotetracycline (AnTc). The bar graphs represent the mean of the reported relative invasion rate data normalized to the inoculum. n=4 biologically independent samples for samples -AnTc and n=10 biologically independent samples for samples +AnTc. Replicates are shown as individual data points and statistical significances were determined by a two-tailed Student's *t* test (** = p<0.01; *** = p<0.001; ns = not significant. For samples -AnTc: *fliC*^{ON} vs. *fliC*^{ON} *ΔfliB*: p=0.0002, *fliC*^{ON} vs. *fliC*^{ON} P_{tet}-*fliB*: p=0.0005, *fliC*^{ON} *ΔfliB* vs. *fliC*^{ON} P_{tet}-*fliB*: ns. For samples +AnTc: *fliC*^{ON} vs. *fliC*^{ON} *ΔfliB*: p<0.0001, *fliC*^{ON} vs. *fliC*^{ON} P_{tet}-*fliB*: ns, *fliC*^{ON}*ΔfliB* vs. *fliC*^{ON} P_{tet}-*fliB*: p=0.0012). (c) Relative *fliB* gene expression of a *fliC*-locked control strain (EM1012), a *fliB* deletion strain (EM4113), and a *fliB*-inducible complementation strain (EM5080) was quantified using qRT-PCR. n=2 biologically independent samples. A representative 1% agarose gel of the amplified *fliB* PCR products is shown below. Source data are provided as a Source Data file.

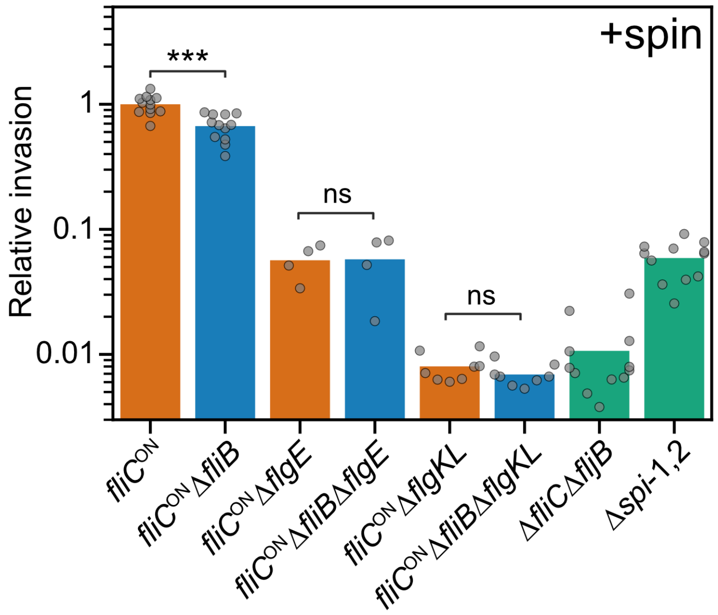

Supplementary Figure 5: Flagellin methylation enhances invasion in a flagella-dependent manner. Infection of MODE-K murine epithelial cells was performed with various flagella assembly mutants at a MOI of 10 for 1h at 37 °C using centrifugation to force contact of the bacteria with the epithelial cells (+spin). Extracellular bacteria were killed by addition of gentamicin for 1 h. Cell lysates were plated in serial dilutions for CFU assessment. Bars represent the mean of the reported relative invasion rate data normalized to the inoculum. n=12 biologically independent samples for *fliC*^{ON}, *fliC*^{ON}Δ*fliB*, Δ*fliC*Δ*fljB*, Δ*spi*-1,2, n=4 biologically independent samples for *fliC*^{ON} Δ*flgE, fliC*^{ON} Δ*fliB* Δ*flgE* and n=8 biologically independent samples for *fliC*^{ON} Δ*flgKL*, *fliC*^{ON} Δ*fliB* Δ*flgKL*. Replicates are shown as individual data points and statistical significances were determined by a two-tailed Student's t test (*** = p<0.001; ns = not significant. *fliC*^{ON} vs. *fliC*^{ON}Δ*fliB*: p<0.0001, *fliC*^{ON} Δ*flgE* vs. *fliC*^{ON} Δ*fliB* Δ*flgE*: ns, *fliC*^{ON} Δ*flgKL* vs. *fliC*^{ON} Δ*fliB* Δ*flgKL*: ns). Source data are provided as a Source Data file.

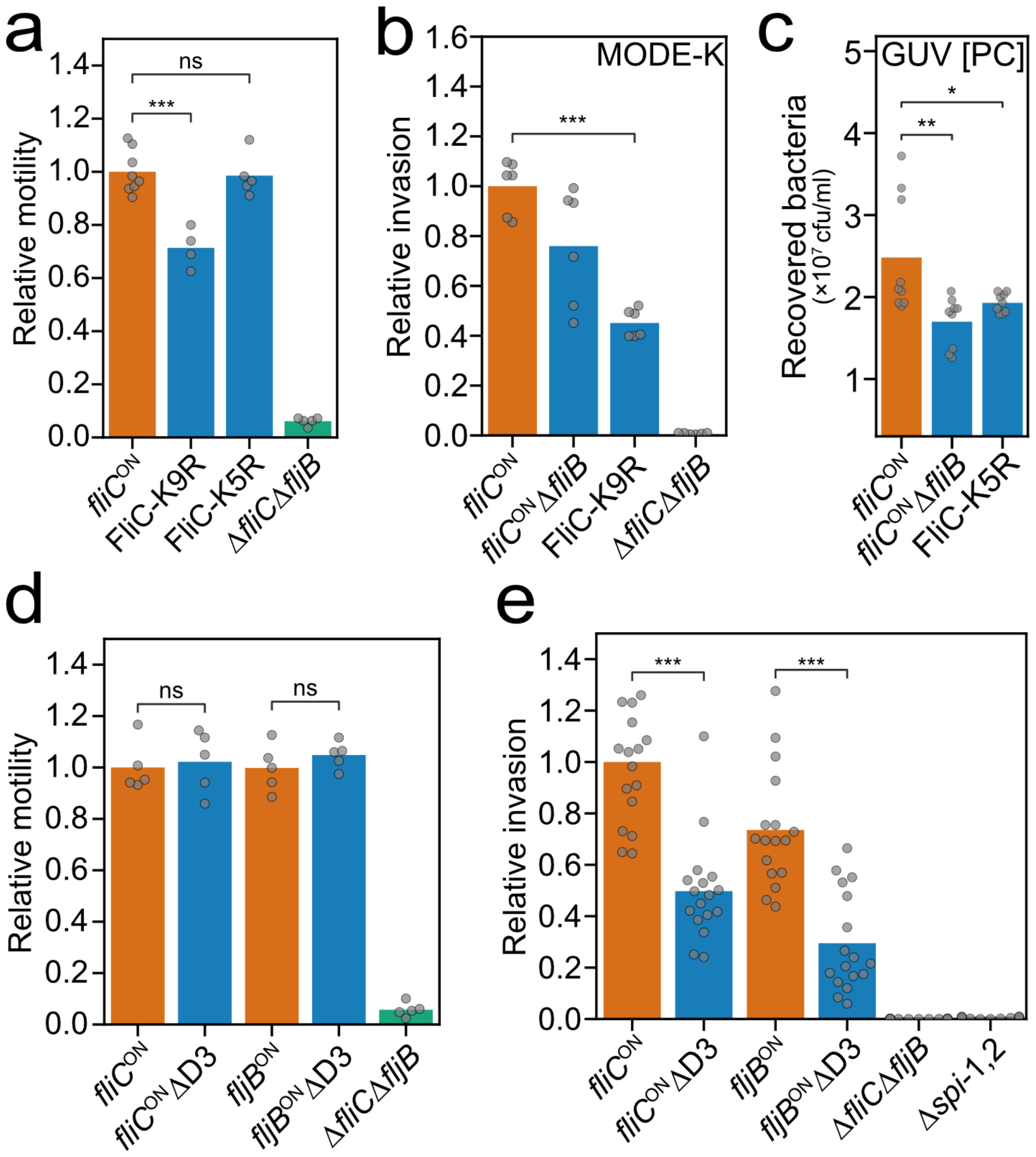

Supplementary Figure 6: Mutating surface-exposed lysines or removing the D3 domain of flagellin affects *Salmonella* Typhimurium epithelial cell invasion. (a) The motility phenotype of chromosomal FliC arginine replacement mutants of surface exposed lysines, FliC-K9R (K204R, K216R, K222R, K229R, K233R, K293R, K309R, K319R, K327R) and FliC-K5R (K204R, K216R, K222R, K229R, K242R), was analyzed using soft-agar plates containing 0.3% agar. n=8 biologically independent samples for *fliC*^{ON}, n=4 biologically independent samples for FliC-K9R, n=5 biologically independent samples for FliC-K5R and Δ*fliC*Δ*fljB*. Replicates are shown as individual data points and statistical significances were determined by a two-tailed Student's t test (*** = p<0.001; ns = not significant. *fliC*^{ON} vs. FliC-K9R: p=0.0001, *fliC*^{ON} vs. FliC-K5R: ns). (b) Invasion of MODE-K murine epithelial cells by the WT expressing FliC, the isogenic Δ*fliB* mutant and an isogenic, chromosomal FliC arginine replacement mutant. Reported are relative invasion rates of MODE-K epithelial cells and invasion was determined as described for

Fig. 4. Contact of the bacteria with the epithelial cells was forced using centrifugation. n=6 biologically independent samples. Replicates are shown as individual data points and statistical significances were determined by a two-tailed Student's t test (*** = p<0.001. *fliC*^{ON} vs. FliC-K9R: p<0.0001). (c) Quantified adhesion of the WT expressing FliC, the isogenic Δ*fliB* mutant and an isogenic, chromosomal FliC arginine replacement mutant (FliC-K5R (K204R, K216R, K222R, K229R, K242R)) to giant unilamellar vesicles (GUV) consisting of 1-palmitoyl-2-oleoyle-glycero-3-phosphocholine (PC). n=9 biologically independent samples. Replicates are shown as individual data points and statistical significances were determined by a two-tailed Student's t test (* = p<0.05; ** = p<0.01. *fliC*^{ON} vs. *fliC*^{ON} Δ*fliB*: p=0.0082, *fliC*^{ON} vs. FliC-K5R: p=0.0365). (d) The motility phenotype of chromosomal FliC and FljB mutants deleted for the respective D3 domain was analyzed using soft-agar plates containing 0.3% agar. n=5 biologically independent samples. Replicates are shown as individual data points and statistical significances were determined by a two-tailed Student's t test (ns = not significant. *fliC*^{ON} vs. *fliC*^{ON} ΔD3: ns, *fljB*^{ON} vs. *fljB*^{ON} ΔD3: ns). (e) Relative invasion rates of MODE-K murine epithelial cells by chromosomal FliC and FljB mutants deleted for the respective D3 domain. Contact of the bacteria with the epithelial cells was forced using centrifugation. The bar graphs represent the mean of the reported relative motility or invasion rate data normalized to the WT or inoculum. n=17 biologically independent samples. Replicates are shown as individual data points and statistical significances were determined by a two-tailed Student's t test (*** = p<0.001. *fliC*^{ON} vs. *fliC*^{ON} ΔD3: *p*<0.0001, *fljB*^{ON} vs. *fljB*^{ON} ΔD3: *p*<0.0001). Source data are provided as a Source Data file.

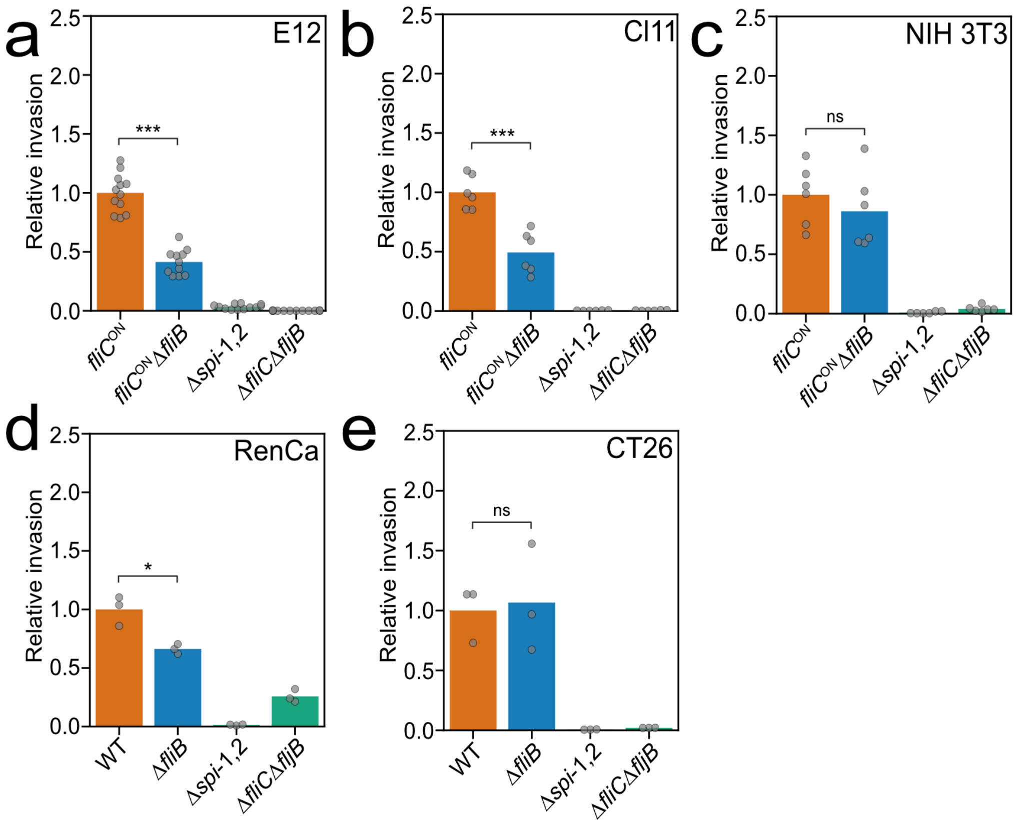

Supplementary Figure 7: Relative invasion rates of different eukaryotic host cell types. (a) Relative invasion of the human epithelial cell line E12 with n=12 biologically independent samples for *fliC*^{ON}, Δ*fliC*Δ*fljB*, Δ*spi*-1,2 and n=11 biologically independent samples for *fliC*^{ON} Δ*fliB*. Replicates are shown as individual data points and statistical significances were determined by a two-tailed Student's t test (*** = $p<0.001$. *fliC*^{ON} vs. Δ*fliC*Δ*fljB*: $p<0.0001$). (b) Relative invasion of the murine epithelial cell line Cl11 with n=6 biologically independent samples. Replicates are shown as individual data points and statistical significances were determined by a two-tailed Student's t test (*** = $p<0.001$. *fliC*^{ON} vs. Δ*fliC*Δ*fljB*: $p=0.0003$). (c) Relative invasion of the murine fibroblast cell line NIH 3T3 with n=6 biologically independent samples. Replicates are shown as individual data points and statistical significances were determined by a two-tailed Student's t test (ns = not significant. *fliC*^{ON} vs. Δ*fliC*Δ*fljB*: ns). (d) Relative invasion of the murine epithelial-like cell line RenCa with n=3 biologically independent samples. Replicates are shown as individual data points and statistical significances were determined by a two-tailed Student's t test (* = $p<0.05$. WT vs. Δ*fljB*: $p=0.0117$). (e) Relative invasion of the murine fibroblast cell line CT26 with n=3 biologically independent samples. Replicates are shown as individual data points and statistical significances were determined by a two-tailed Student's t test (ns = not significant. WT vs. Δ*fljB*: ns). Eukaryotic cells were infected with *Salmonella* Typhimurium flagella methylation mutants as described for Fig. 4. The bar graphs represent the mean of the reported relative invasion rate data normalized to the inoculum. Source data are provided as a Source Data file.

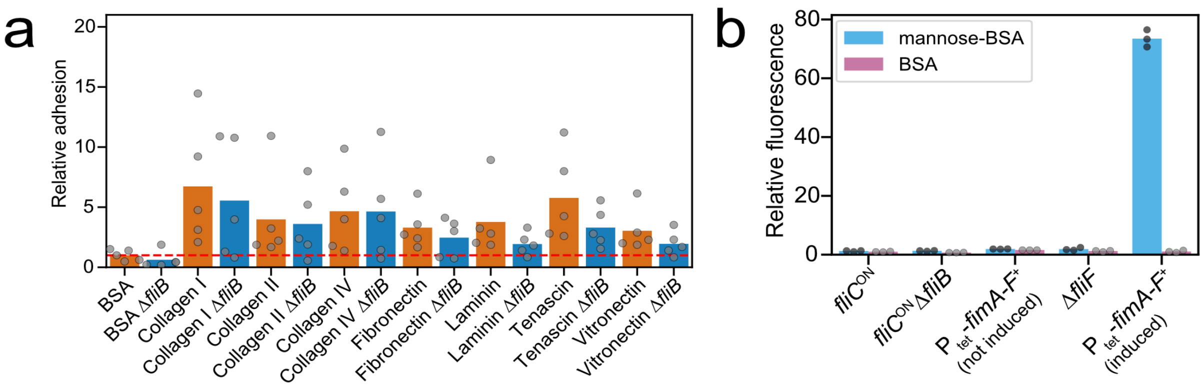

Supplementary Figure 8: Flagella methylation does not mediate binding of *Salmonella* to extracellular matrix proteins or mannose residues. (a) Adhesion of *S. enterica* to extracellular matrix (ECM) proteins *in vitro*. Control and Δ*fliB* strains were added to ECM protein pre-coated 96-well plates and incubated for 1h at 37 °C. After extensive washing, adherent bacteria were plated in serial dilutions for CFU assessment. n=5 biologically independent samples. (b) Methylated flagella do not contribute to binding of *Salmonella* to surface-exposed mannose. Plastic surfaces were coated with BSA or mannose-BSA and strains harboring pFU228-P_{gapdh}-mCherry were added for 1 h at 37 °C. After washing, fluorescence was measured in a microplate reader and values were normalized to BSA. P_{tet}-*fimA-F* served as positive control after induction with 100 ng·mL^{-1} anhydrotetracycline. The bar graphs represent the mean of the reported data and replicates are shown as individual data points. n=3 biologically independent samples. Source data are provided as a Source Data file.

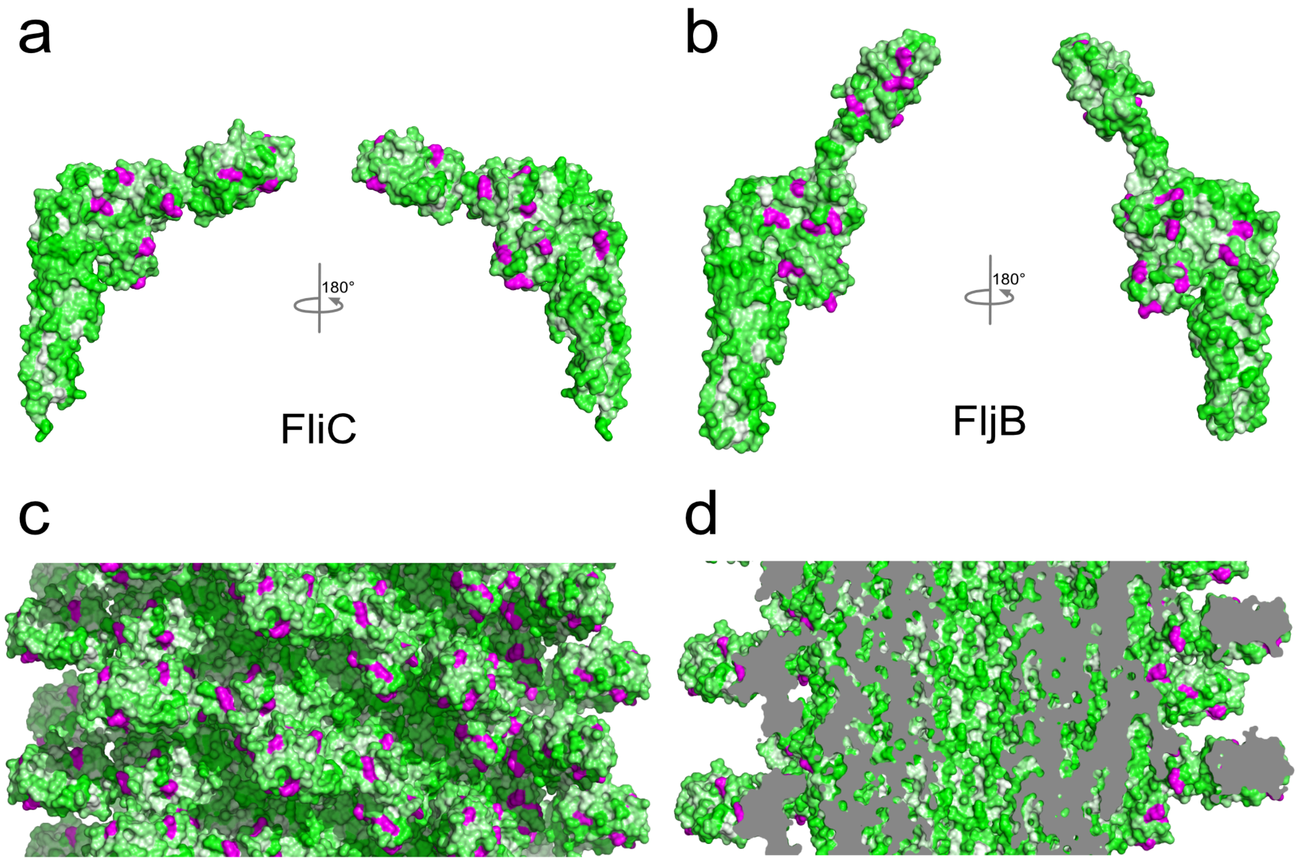

Supplementary Figure 9: Surface hydrophobicity distribution of flagellins and the flagellar filament. Connolly surface representation of the flagellins FliC (a) and FljB (b). Surface color represents the surface hydrophobicity according to the Eisenberg scale[10] (green to white indicates increasing hydrophobicity) and methylated lysine residues are highlighted in magenta. Surface hydrophobicity distribution of the outer (c) and the inner surface (d) of the FliC flagellar filament[11] with methylation sites highlighted in magenta.

Supplementary Figure 10: Uncropped original blots and gels

Figure 3a

55 kDa

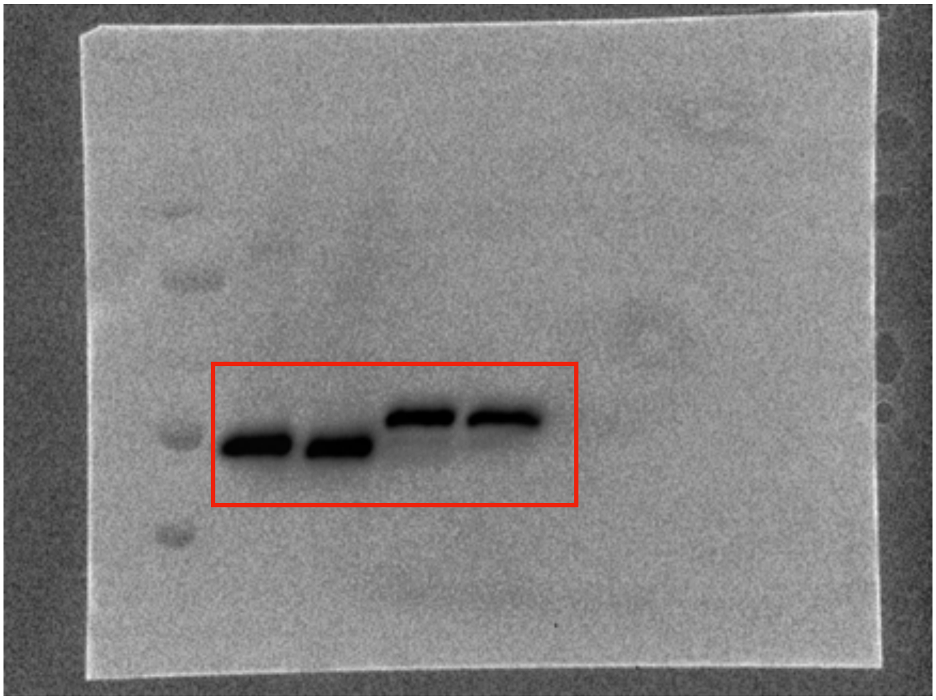

Supplemental Figure 4c

500 bp
200 bp
100 bp

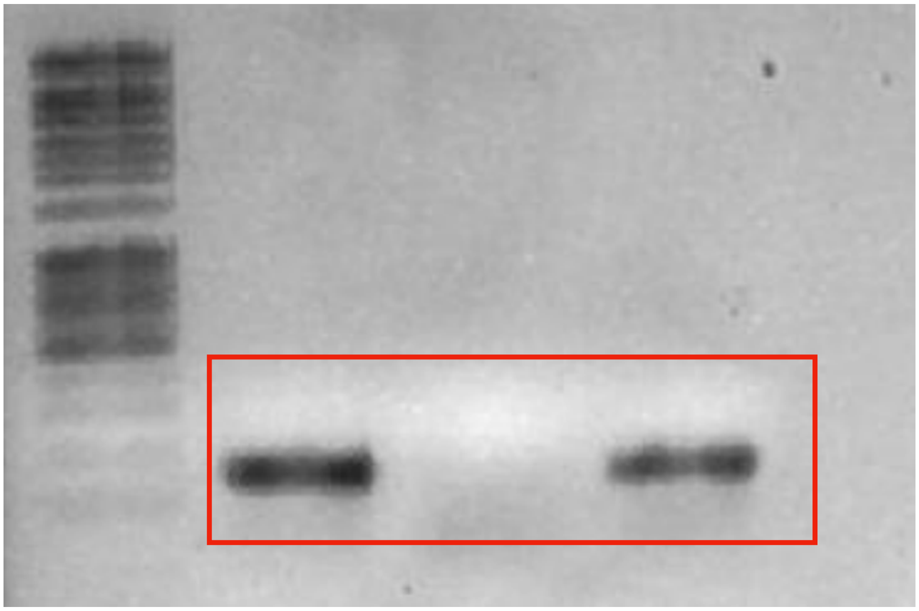

Supplementary Tables:

Supplementary Table 1: Data collection and refinement statistics for FljB$_{55\text{-}462}$.

**Data collection**	
Space group	*C*2
Cell dimensions	
a, b, c (Å)	97.47, 38.28, 124.69
β (°)	103.54
Resolution (Å)	50.00 – 2.00 (2.05 – 2.00)
R_{sym} or R_{merge}	0.096 (0.673)
$I/\sigma(I)$	11.25 (2.26)
Completeness (%)	98.7 (86.0)
Redundancy	5.56 (4.65)

**Refinement**	
Resolution (Å)	48.11 – 2.00
No. reflections	30567
R_{work}/R_{free}	0.213 / 0.259
No. atoms	
Protein	2938
Water	299
B-factors	
Protein	34.63
Water	35.98
R.m.s. deviations	
Bond lengths (Å)	0.008
Bond angles (°)	1.3
Ramachandran plot	
Favored	98.0 %
Allowed	1.5 %
Outliers	0.5 %

Values in parentheses are for highest-resolution shell. R_{free} is calculated using 5 % of reflections randomly chosen.

Supplementary Table 2: ScanProsite normalized scores of methylation sites matching the Big-1 domain profile.

Site	Sequence	Score
FljB 2	LDDAAI**K**AATGGT	4.107
FljB 3	VTGGAV**K**FDADNN	4.541
FljB 5	TGADAA**K**NGDYEV	4.293
FljB 6	LAAGAT**K**TTMPAG	4.396
FljB 7	VVSADA**K**NALIAG	4.603
FljB 8	NGAELV**K**MSYTDK	4.128
FljB 12	TGAIKA**K**TTSYTA	4.706
FljB 13	AADGTT**K**TAANQL	4.892
FljB 14	LGGVDG**K**TEVVTI	4.293
FljB 18	LAEAAA**K**TTENPL	4.582
FliC 1	LDNSTF**K**ASATGL	5.223
FliC 4	FDDTTG**K**YYAKVT	5.429
FliC 5	VTGGTG**K**DGYYEV	5.140
FliC 6	ADLTEA**K**AALTAA	4.933
FliC 7	GTASVV**K**MSYTDN	4.231
FliC 9	YSATQN**K**DGSISI	4.995
FliC 10	ISINTT**K**YTADDG	4.272
FliC 11	ADDGTS**K**TALNKL	4.417
FliC 13	LGGADG**K**TEVVSI	4.148
FliC 16	TENPLQ**K**IDAALA	4.128

The table includes only the sites matching the Big-1 domain profile. A sequence of 13 residues was scanned for each methylation site. The methylated lysine at the center of each sequence is indicated in bold. Numbering of the sites refers to the position in the sequences reported in Fig. 1d. The normalized score reported by ScanProsite measures the confidence level. All the sites fall into the "twilight zone" of confidence (score between 4.1 and 8.5) where true and false positive can co-exist.

Supplementary Table 3: *Salmonella enterica* serovar Typhimurium SL1344 strains used in this study.

Strains	Relevant characteristics	Reference/source
EM774	SL1344 WT	Lab collection
EM812	SL1344 Δ*fliC7716* Δ*fljB22314*	Lab collection
EM949	SL1344 Δ*hin-5717*::FCF (*fliC*^{ON})	Lab collection
EM1012	SL1344 Δ*hin-5717*::FRT (*fliC*^{ON})	Lab collection
EM1013	SL1344 Δ*hin-5718*::FRT (*fljB*^{ON})	Lab collection
EM1022	SL1344 Δ*invH-sprB*::FRT (Δ*spi-1*) Δ*sseA-ssaU*::FCF (Δ*spi-2*)	[8]
EM1398	SL1344 Δ*hin-5717*::FRT (*fliC*^{ON}) Δ*att*P22::FKF	[8]
EM3122	SL1344 Δ*invH-sprB*::FKF (Δ*spi-1*)	This study
EM3123	SL1344 Δ*hin-5717*::FRT (*fliC*^{ON}) Δ*invH-sprB*::FKF (Δ*spi-1*)	[8]
EM3124	SL1344 Δ*hin-5718*::FRT (*fljB*^{ON}) Δ*invH-sprB*::FKF (Δ*spi-1*)	[8]
EM3734	SL1344 Δ*fliB8191*	This study
EM3758	SL1344 Δ*fliB8191* Δ*invH-sprB*::FKF (Δ*spi-1*)	This study
EM3743	SL1344 Δ*hin-5717*::FCF (*fliC*^{ON}) Δ*fliB8191*	This study
EM3759	SL1344 Δ*fliB8191* Δ*hin-5717*::FCF (*fliC*^{ON}) Δ*invH-sprB*::FKF (Δ*spi-1*)	This study
EM3760	SL1344 Δ*fliB8191* Δ*hin-5718*::FCF (*fljB*^{ON}) Δ*invH-sprB*::FKF (Δ*spi-1*)	This study
EM4113	SL1344 Δ*fliB8191* Δ*hin-5717*::FRT (*fliC*^{ON})	This study
EM4114	SL1344 Δ*fliB8191* Δ*hin-5718*::FRT (*fljB*^{ON})	This study
EM4412	SL1344 Δ*hin-5717*::FCF Δ*fljB5631*::FRT Δ*fliC22815* (ΔD3)	This study
EM4414	SL1344 Δ*hin-5718*::FRT Δ*fliC7716* Δ*fljB22816* (ΔD3)	This study
EM4660	SL1344 Δ*fliB8191* Δ*hin-5717*::FRT (*fliC*^{ON}) Δ*att*P22::FCF	This study
EM4699	SL1344 Δ*hin-5717*::FRT (*fliC*^{ON}) Δ*flgE6231*(Δaa R106-Q112)::*tetRA*	This study
EM4700	SL1344 Δ*hin-5717*::FRT (*fliC*^{ON}) Δ*fliB8191* Δ*flgE6231*(Δaa R106-Q112)::*tetRA*	This study
EM4934	SL1344 Δ*motAB*::*tetRA*	Lab collection
EM4949	SL1344 Δ*hin-5717*::FRT (*fliC*^{ON}) Δ*flgKL5739*::FKF	This study
EM4950	SL1344 Δ*hin-5717*::FRT (*fliC*^{ON}) Δ*fliB8191* Δ*flgKL5739*::FKF	This study
EM5080	SL1344 *fliB22903*::Tn*10d*Tc (*tetR* terminator) Δ*hin-5717*::FCF (*fliC*^{ON})	This study
EM5627	SL1344 Δ*hin-5717*::FCF Δ*fljB5631*::FRT *fliC22942* (K204R, K216R, K222R, K229R, K233R, K293R, K309R, K319R, K327R)	This study
EM7748	SL1344 P_{tetA}-*fimAICDHF* / pFU228 (P_{gapdh}-*mCherry*)	This study
EM7816	SL1344 Δ*hin-5717*::FRT (*fliC*^{ON}) / pFU228 (P_{gapdh}-*mCherry*)	This study
EM7817	SL1344 Δ*hin-5717*::FRT (*fliC*^{ON}) Δ*fliB8191* / pFU228 (P_{gapdh}-*mCherry*)	This study
EM7850	SL1344 Δ*fliF5629*::FKF / pFU228 (P_{gapdh}-*mCherry*)	This study
EM7842	SL1344 Δ*fliB8191* Δ*motAB*::*tetRA*	This study
EM9818	SL1344 Δ*hin-5717*::FCF *fliC23290* (K204R, K216R, K222R, K229R, K242R)	This study

Supplementary References:

1. Wilson, D. R. & Beveridge, T. J. Bacterial flagellar filaments and their component flagellins. *Can J Microbiol* **39**, 451-472 (1993).
2. Beatson, S. A., Minamino, T. & Pallen, M. J. Variation in bacterial flagellins: from sequence to structure. *Trends Microbiol* **14**, 151-155 (2006).
3. Evans, L. D., Poulter, S., Terentjev, E. M., Hughes, C. & Fraser, G. M. A chain mechanism for flagellum growth. *Nature* **504**, 287-290 (2013).
4. Josenhans, C. & Suerbaum, S. The role of motility as a virulence factor in bacteria. *Int J Med Microbiol* **291**, 605-614 (2002).
5. Hayashi, F. et al. The innate immune response to bacterial flagellin is mediated by Toll-like receptor 5. *Nature* **410**, 1099-1103 (2001).
6. Reid, S. D., Selander, R. K. & Whittam, T. S. Sequence diversity of flagellin (*fliC*) alleles in pathogenic *Escherichia coli*. *J Bacteriol* **181**, 153-160 (1999).
7. Samatey, F. A. et al. Structure of the bacterial flagellar protofilament and implications for a switch for supercoiling. *Nature* **410**, 331-337 (2001).
8. Horstmann, J. A. et al. Flagellin phase-dependent swimming on epithelial cell surfaces contributes to productive *Salmonella* gut colonisation. *Cell Microbiol* **19**, e12739 (2017).
9. Carvalho, P. C. et al. Integrated analysis of shotgun proteomic data with PatternLab for proteomics 4.0. *Nat Protoc* **11**, 102-117 (2016).
10. Eisenberg, D., Schwarz, E., Komaromy, M. & Wall, R. Analysis of membrane and surface protein sequences with the hydrophobic moment plot. *J Mol Biol* **179**, 125-142 (1984).
11. Maki-Yonekura, S., Yonekura, K. & Namba, K. Conformational change of flagellin for polymorphic supercoiling of the flagellar filament. *Nat Struct Mol Biol* **17**, 417-422 (2010).
