# Supplementary material for: Methylation of Salmonella Typhimurium flagella promotes bacterial adhesion and host cell invasion
Source: Nat Commun. 2020 Apr 24;11:2013. doi: 10.1038/s41467-020-15738-3 (PMC7181671; doi:10.1038/s41467-020-15738-3)
Supplement: Supplementary file 3 — Reporting Summary [file 41467_2020_15738_MOESM3_ESM.pdf]

nature research

Corresponding author(s): Dr. Marc Erhardt, Dr. Michael Kolbe

Last updated by author(s): Mar 9, 2020

Reporting Summary

Nature Research wishes to improve the reproducibility of the work that we publish. This form provides structure for consistency and transparency in reporting. For further information on Nature Research policies, see Authors & Referees and the Editorial Policy Checklist .

Statistics

For all statistical analyses, confirm that the following items are present in the figure legend, table legend, main text, or Methods section.

n/a	Confirmed	
☐	☒	The exact sample size (n) for each experimental group/condition, given as a discrete number and unit of measurement
☒	☐	A statement on whether measurements were taken from distinct samples or whether the same sample was measured repeatedly
☐	☒	The statistical test(s) used AND whether they are one- or two-sided<br>*Only common tests should be described solely by name; describe more complex techniques in the Methods section.*
☒	☐	A description of all covariates tested
☒	☐	A description of any assumptions or corrections, such as tests of normality and adjustment for multiple comparisons
☐	☒	A full description of the statistical parameters including central tendency (e.g. means) or other basic estimates (e.g. regression coefficient) AND variation (e.g. standard deviation) or associated estimates of uncertainty (e.g. confidence intervals)
☐	☒	For null hypothesis testing, the test statistic (e.g. F, t, r) with confidence intervals, effect sizes, degrees of freedom and P value noted<br>*Give P values as exact values whenever suitable.*
☒	☐	For Bayesian analysis, information on the choice of priors and Markov chain Monte Carlo settings
☒	☐	For hierarchical and complex designs, identification of the appropriate level for tests and full reporting of outcomes
☒	☐	Estimates of effect sizes (e.g. Cohen's d, Pearson's r), indicating how they were calculated

Our web collection on statistics for biologists contains articles on many of the points above.

Software and code

Policy information about availability of computer code

Data collection	Helmholtz-Zentrum Berlin für Materialien und Energie. (2016). The MX Beamlines BL14.1-3 at BESSY II. Journal of large-scale research facilities, 2, A47. http://dx.doi.org/10.17815/jlsrf-2-64
Data analysis	GraphPad Prism 6.0h (https://www.graphpad.com)<br>statannot 0.1.0, a Python package for statistical annotations and analyses (https://github.com/webermarcolivier/statannot)<br>UCSF Chimera 1.13.1<br>PyMol 2.2.3<br>ESPript version 3.0 (http://espript.ibcp.fr)<br>ProteomeDiscoverer 2.0 (Thermo Fisher Scientific)<br>PatterLab for proteomics 4.0<br>Phaser version 2.5.2<br>XDS version 6.12.2010<br>Coot version 0.8.9.1<br>CNS version 1.3<br>PDBeFold version 2.59<br>NIH ImageJ 1.48v<br>XCalibur Software 3.0

For manuscripts utilizing custom algorithms or software that are central to the research but not yet described in published literature, software must be made available to editors/reviewers. We strongly encourage code deposition in a community repository (e.g. GitHub). See the Nature Research guidelines for submitting code & software for further information.

Data

Policy information about availability of data

All manuscripts must include a data availability statement. This statement should provide the following information, where applicable:

- Accession codes, unique identifiers, or web links for publicly available datasets
- A list of figures that have associated raw data
- A description of any restrictions on data availability

The data that support the findings of this study are available from the corresponding authors upon request. Methylation data as well as corresponding MS raw data have been deposited to the ProteomeXchange Consortium via the PRIDE partner repository with the dataset identifier. The coordinates of the flagellin FljB have been deposited in the worldwide Protein Data Bank (wwPDB) under accession number 6RGV [http://doi.org/10.2210/pdb6rgv/pdb].

Field-specific reporting

Please select the one below that is the best fit for your research. If you are not sure, read the appropriate sections before making your selection.

☒ Life sciences ☐ Behavioural & social sciences ☐ Ecological, evolutionary & environmental sciences

For a reference copy of the document with all sections, see nature.com/documents/nr-reporting-summary-flat.pdf

Life sciences study design

All studies must disclose on these points even when the disclosure is negative.

Sample size	No sample size was calculated. Sample sizes were chosen according to our experience in similar experimental setups (e.g. Horstmann, J. A. et al. Flagellin phase-dependent swimming on epithelial cell surfaces contributes to productive Salmonella gut colonisation. Cell Microbiol 19, e12739 (2017)).
Data exclusions	No data was excluded from the analysis.
Replication	All reported experiments have been repeated at least 2 times with independent samples. All experimental results shown were reproducible. No exclusion criteria were pre-established.
Randomization	No experimental groups were formed/compared. Data was collected randomly in each set of experiments.
Blinding	The researchers were not blinded to sample identity.

Reporting for specific materials, systems and methods

We require information from authors about some types of materials, experimental systems and methods used in many studies. Here, indicate whether each material, system or method listed is relevant to your study. If you are not sure if a list item applies to your research, read the appropriate section before selecting a response.

Materials & experimental systems

n/a	Involved in the study	
☐	☒	Antibodies
☐	☒	Eukaryotic cell lines
☒	☐	Palaeontology
☐	☒	Animals and other organisms
☒	☐	Human research participants
☒	☐	Clinical data

Methods

n/a	Involved in the study	
☒	☐	ChIP-seq
☒	☐	Flow cytometry
☒	☐	MRI-based neuroimaging

Antibodies

Antibodies used	anti-FliC: Difco Salmonella H Antiserum I Difco 228241; anti-FljB: Difco Salmonella H Antiserum Single Factor 2 Difco 224741; Bio-Rad Immun-Star Goat Anti-Rabbit (GAR)-HRP Conjugate, catalog number 170-5046; anti-rabbit Alexa-Fluor488, Invitrogen, catalog number A-11094.
Validation	Antibodies were validated in our lab using Western blot against whole-cell lysates obtained from suitable Salmonella enterica serovar Typhimurium deletion mutants (ΔfliC for anti-FliC and ΔfljB for anti-FljB).

Eukaryotic cell lines

Policy information about cell lines

Cell line source(s)	The murine epithelial cell lines MODE-K (K. Vidal, I. Grosjean, J. P. evillard, C. Gespach, D. Kaiserlian, J Immunol Methods 166, 63 (1993)), the murine epithelial-like cell line Renca (CRL-2947), the human epithelial cell line HT29-MTX-E12 (E12) (N. Navabi, M. A. McGuckin, S. K. Lindén, PLoS One 8, e68761 (2013)), and the mouse fibroblast cell lines NIH-3T3 (CRL-1658) and CT26 (CRL-2638) were used in this study. The muGob (Cl11) cell line was obtained from InSCREENeX GmbH, Germany.
Authentication	None of the cell lines were authenticated.
Mycoplasma contamination	The cell lines were regularly tested negative for mycoplasma contamination.
Commonly misidentified lines (See ICLAC register)	No commonly misidentified lines were used.

Animals and other organisms

Policy information about studies involving animals; ARRIVE guidelines recommended for reporting animal research

Laboratory animals	7-week-old C57BL/6 mice were purchased from Janvier Laboratories. Mice were housed in individually-ventilated cages with free access to autoclaved water and chow.
Wild animals	No wild animals were used in this study.
Field-collected samples	No field-collected samples were used in this study.
Ethics oversight	All animal experiments were performed according to guidelines of the German Law for Animal Protection and with permission of the local ethics committee and the local authority LAVES (Niedersächsisches Landesamt für Verbraucherschutz und Lebensmittelsicherheit) under permission number 33.19-42502-04-13/1191.

Note that full information on the approval of the study protocol must also be provided in the manuscript.
